# Precision antimicrobial therapeutics: the path of least resistance?

**DOI:** 10.1038/s41522-018-0048-3

**Published:** 2018-02-27

**Authors:** Caitlin N. Spaulding, Roger D. Klein, Henry L. Schreiber, James W. Janetka, Scott J. Hultgren

**Affiliations:** 1 Department of Immunology and Infectious Disease, Harvard T.H. Chan School of Public Health, Boston, MA 02115 USA; 2 Department of Molecular Microbiology, Washington University School of Medicine, St. Louis, MO 63110 USA; 3 Center for Women’s Infectious Disease Research (CWIDR), Washington University School of Medicine, St. Louis, MO 63110 USA; 4 Department of Biochemistry and Molecular Biophysics, Washington University School of Medicine, St. Louis, MO 63110 USA

## Abstract

The emergence of drug-resistant pathogens has led to a decline in the efficacy of traditional antimicrobial therapy. The rise in resistance has been driven by widespread use, and in some cases misuse, of antibacterial agents in treating a variety of infections. A growing body of research has begun to elucidate the harmful effects of broad-spectrum antibiotic therapy on the beneficial host microbiota. To combat these threats, increasing effort is being directed toward the development of precision antimicrobial therapeutics that target key virulence determinants of specific pathogens while leaving the remainder of the host microbiota undisturbed. This includes the recent development of small molecules termed “mannosides” that specifically target uropathogenic *E. coli* (UPEC). Mannosides are glycomimetics of the natural mannosylated host receptor for type 1 pili, extracellular appendages that promotes UPEC colonization in the intestine. Type 1 pili are also critical for colonization and infection in the bladder. In both cases, mannosides act as molecular decoys which potently prevent bacteria from binding to host tissues. In mice, oral treatment with mannosides simultaneously clears active bladder infection and removes intestinal UPEC while leaving the gut microbiota structure relatively unchanged. Similar treatment strategies successfully target other pathogens, like adherent-invasive *E. coli* (AIEC), an organism associated with Crohn’s disease (CD), in mouse models. While not without its challenges, antibiotic-sparing therapeutic approaches hold great promise in a variety of disease systems, including UTI, CD, otitis media (OM), and others. In this perspective we highlight the benefits, progress, and roadblocks to the development of precision antimicrobial therapeutics.

## Introduction

Antibiotics are considered the standard of care for the treatment of most bacterial infections caused by drug-susceptible organisms. However, the worldwide spread of drug-resistant bacterial pathogens has greatly limited the repertoire of antibiotics available to effectively treat patients. As a result, clinicians are becoming increasingly reliant on last-line antimicrobial agents to treat a growing number of common bacterial infections. The efficacy of these agents has also begun to decline in the face of rapidly evolving resistant bacterial populations. Additionally, a growing number of studies are finding that alterations to the community structure of the host commensal microbiota following treatment with traditional antibiotics can have negative effects on long-term host health, especially when administered during childhood.

A recent review on antimicrobial resistance led by the British government has suggested that, without a rapid expansion of our antimicrobial arsenal, “superbugs” resistant to existing antibiotics could kill more than 10 million people a year by 2050.^1^ Further, research from the World Bank suggests that the dramatic increase in antibiotic-resistant infections could have dire consequences on the world economy with an estimated $100 trillion being spent to combat these infections by 2050.^2^ Together, these findings highlight the antibiotic resistance crisis we currently face and underscore the need to develop treatments that can potentiate or replace broad-spectrum antibiotic therapy in patients with bacterial infections. To address this emerging crisis, it is essential that novel treatments be developed to spare existing broad-spectrum antibiotics and selectively target and remove an infecting pathogen while leaving the community structure of the surrounding microbiota unchanged. These new therapeutics, hitherto referred to as precision antimicrobials, include both anti-virulence compounds that inhibit bacterial pathogenesis and persistence, as well as new compounds that are bactericidal or bacteriostatic to a minimal number of bacterial pathogens.

In this perspective, we explore the differences between broad-spectrum antibiotics and precision antimicrobial therapies and highlight the benefits and challenges of developing precision therapeutics. Further, we highlight a series of potential applications for these precision antimicrobials.

## The mutualistic symbiotic relationship between host and microbiota

All mammals harbor complex and dynamic populations of microorganisms (known as the microbiota), which are made up of bacteria, archea, fungi, viruses, and protozoa^3^ and colonize mucosal surfaces, such as those found in the nose, mouth, airways, gut, and urogenital tract, as well as non-mucosal surfaces, like the skin. However, the intestine holds the largest collection of microbes, with a total of ~10^14^ bacterial cells. The collection of bacterial genomes in the microbiota contains >5 million genes, outnumbering the number of human genes by orders of magnitude.^4^

Humans and their associated microbial communities have co-evolved, as a recent study shows that over the last 15 million years intestinal bacteria have co-speciated with hominids.^5^ This study examined two highly conserved clades of intestinal bacteria, Bacteroidaceae and Bifodobacteriaceae, in humans, chimpanzees, and gorillas, and found that the estimated time of divergence between microbial communities in hominid species occurred around the same time as the presumed hominid speciation event.^5^ This work suggests that different evolutionary pressures faced by humans and apes likely shaped both host physiology and their associated microbial communities. Thus, the human microbiota has influenced and been influenced by the evolution of humans based on critical mutualistic symbiotic relationships. For example, microbial communities benefit from the abundance of nutrients and space available within the host and thus have evolved strategies to maintain their association with the host. In return, these organisms play essential roles in host development and health. During early life, host–microbiota interactions influence the development of the host immune system and have been implicated in muscle, adipose tissue, and bone growth.^6^ The microbiota provides additional benefits throughout the lifetime of the host, including the synthesis of vitamins that promote host health and the liberation of otherwise inaccessible nutrients from the host diet.^3^ Further, by sequestering available nutrients and occupying space within the host, the residing microbiota promotes colonization resistance and thus discourages colonization and subsequent infection by pathogenic organisms.

While the interactions between the host and microbiota are generally mutualistic, disruption of this relationship can occur, leading to a state of dysbiosis. During this period of imbalance, species that typically dominate the community become underrepresented while low-abundance species that were restricted in growth are enabled to expand their population to fill the void. Dysbiosis can occur when a pathogenic organism infiltrates a community and seizes space and nutrients from commensal organisms. In healthy individuals, the host immune system, by mechanism(s) that are under investigation by a number of groups, tolerates the presence of a commensal microbiota.^7,8^ During periods of infection or dysbiosis, the immune system attempts to restore balance by removing the pathogen and restoring the community structure through inflammation and secretion of antimicrobial peptides among other responses. Yet, in some cases, an over-exuberant or continuous immune response further exacerbates the dysbiotic condition, generating a vicious cycle that results in a protracted imbalance in the gut microbiota. Dysbioses, particularly those that last for extended periods of time, are associated with a number of human diseases and infection, including inflammatory bowel disease (IBD), urinary tract infections (UTI), otitis media (OM), sinusitis, conjunctivitis, and acne.

## The consequences of antibiotic exposure

While it is undeniable that antibiotic therapy is an invaluable clinical tool, an increasing number of studies have demonstrated that perturbations to the gut microbiota by oral broad-spectrum antibiotic treatment results in alterations to the functions of the microbiota in ways that are ultimately detrimental to host health. For example, disrupting the gut microbiota with broad-spectrum antibiotics during childhood may alter the development of a child’s immune system as well as the growth of adipose, muscle, and bone tissues.^6^ Broad-spectrum antibiotic exposure also increases the spread and uptake of bacterial genetic elements, including plasmids encoding antibiotic resistance genes, thus contributing to the development and spread of antibiotic resistance while selecting for the growth of bacteria that are resistant to the antibiotic being consumed.^9^ Further, by altering the community structure of the microbiota, broad-spectrum antibiotics also disrupt colonization resistance, opening space for pathogens to colonize or for typically low-abundance organisms present in the community to bloom and cause infection within the gut and/or at extra-intestinal sites which can result in long-lasting dysbiosis.

The bloom of normally restricted organisms is observed after treatment of mice with a single oral dose of the broad-spectrum antibiotic streptomycin, which produces high levels of intestinal inflammation and enhances colonization by *E. coli* species.^10–12^ The increased fitness of *E. coli* may be due to several factors, such as disruption of colonization resistance and alterations in the generation of cellular energy. Previous work has found that nitrate, released into the gut lumen as a byproduct of the streptomycin-induced intestinal inflammatory response, can be used by *E. coli* as a terminal electron acceptor for anaerobic respiration, a process not available to many strict anaerobes present in the gut that lack the necessary nitrate/nitrite reductase enzymes.^10,13–15^ High levels of intestinal inflammation have also been linked to increases in *E. coli* colonization of the gut of patients with IBD. IBD represents a subset of syndromes that are characterized by constitutively high levels of intestinal inflammation. Biopsy specimens from patients with Crohn’s disease (CD) and Ulcerative Colitis, two IBD syndromes, revealed that these patients have a 3–4 log increase in the levels of Enterobacteriaceae in their intestines compared to healthy controls.^16^ The enhanced fitness of *E. coli* during intestinal inflammation may also increase patients’ chances of having bladder infection as several clinical studies have found that IBD patients have a significantly increased risk of recurrent UTI (rUTI) with >80% of patients having rUTI.^17,18^

Therefore, even when used to target susceptible pathogens, treatment with broad-spectrum antibiotics that affect a larger proportion of a community may be detrimental to host health. Therefore, the need to develop highly targeted, precision therapeutics that can specifically kill or eliminate antibiotic-resistant pathogens while producing minimal changes to the community structure of the microbiota has gained increased urgency.

## Developing precision treatments for UTIs

Uropathogenic *E. coli* (UPEC) are normal components of the gut microbiota; however, when shed in the feces UPEC can colonize peri-urethral or vaginal tissue before ascending through the urethra and accessing the bladder, causing UTI.^19–21^ Although stochastic in nature, the frequency of UPEC shedding in feces and subsequent migration to the bladder is thought to be related to the gastrointestinal UPEC burden. Thus, a bloom in *E. coli* levels during periods of intestinal dysbiosis result in greater levels of UPEC shedding into feces and a concomitant increase in the rate of UPEC transmission to the bladder.^17,18^ The standard of care for individuals with UTI is antibiotic therapy. However, the observation that 20–40% of women will have one or more recurrences within months of her initial UTI despite appropriate antibiotic therapy suggests that broad-spectrum antibiotics have limited long-term efficacy for UTI treatment.^22^ This problem is compounded by the rising incidence of antibiotic-resistant uropathogens; the prevalence of UPEC strains resistant to fluoroquinolones, a so-called “last-line” antibiotic for UTI treatment, has reached up to 70% in countries such as India, China, and Vietnam and up to 50% in some European countries.^23^ The resultant reliance on carbepenems to treat an increasing number of patients with drug-resistant UTI has, in turn, driven the development and expansion of carbapenem-resistant Enterobacteriaceae (CRE). Increased incidence of CRE is particularly concerning as several reports have associated these infections with up to 50% mortality.^24,25^ The identification of a UTI caused by a CRE strain that is also resistant to colistin, a drug of last resort for treating CRE resistance isolates, in a woman from the United States emphasizes the urgent and alarming nature of the antibiotic resistance crisis we currently face.^23,26–28^ The drug resistance index (DRI) is a composite measure that combines the ability of antibiotics to treat infections with the extent of their use in clinical practice. The DRI provides an aggregate trend measure of the effectiveness of available drugs. The index for UTIs shows the number of infections facing treatment difficulties has been increasing since the mid-2000s due to the rapid spread of resistance among UPEC, underscoring the need to develop new therapeutics geared toward the emerging threat of drug-resistant Gram-negative organisms.^29,30^

A critical stage of UPEC pathogenesis is colonization of host tissue. To accomplish this, UPEC express chaperone usher pathway (CUP) pili. Single UPEC strains can carry between 5 and 16 distinct CUP pilus operons, many of which are known to aid UPEC strains in their colonization of many different host habitats, including the gut, vagina, urethra, bladder, and kidneys.^31^ The carriage of these gene clusters is not uniform, as some CUP pili are carried by almost every *E. coli* strain while others are carried only by a minority of strains. The expression of CUP pili is tightly regulated, resulting in the expression of only one pilus type at a time.^32^ CUP pili are tipped with adhesins that mediate UPEC tropism by binding distinct ligand(s) with stereochemical specificity. Adhesins are two-domain proteins made up of an N-terminal lectin domain, which is responsible for recognition and attachment to specific ligands, and a C-terminal pilin domain that connects the adhesin to the bulk of the pilus.^33^ The availability of the ligands bound by adhesins is vital to infection and the presence of these ligands differs between body sites/habitats. Multiple pilus types have been shown to promote UPEC colonization of the urinary tract.

In mice, type 1 pili tipped with the FimH adhesin mediate acute bladder colonization by binding to mannosylated proteins expressed on the surface of bladder epithelial cells.^34^ A complete *fim* operon encoding the necessary components of the type 1 pilus is carried by approximately 75% of *E. coli* and is further enriched in UPEC strains.^27^ FimH-mediated binding to the bladder can lead to UPEC invasion into luminal bladder epithelial cells where they replicate to high levels while protected from many host defenses, thus promoting ongoing infection. Clinical studies have implicated type 1 pili as a critical colonization factor during UTI in women.^35,36^ CUP pili also promote UPEC colonization in the gut reservoir. A recent study found that type 1 and F17-like pili promote the establishment and/or maintenance of the UPEC intestinal reservoir in a streptomycin-treated mouse model of intestinal colonization.^12^ Interestingly, the purified lectin domains of the type 1 and F17-like adhesins (FimH and UclD, respectively) were shown to bind to distinct micro-habitats within the colonic crypt. FimH bound to the more differentiated cells in the upper crypts and in the surface epithelial cuffs that line the intestinal lumen. In contrast, UclD bound the lower, less differentiated cells of the crypts.^12^ Strikingly, phylogenetic analysis of F17-like pili suggest that this system was acquired from another species of gut-colonizing bacteria, suggesting that UPEC acquired the system to enhance colonization of a habitat within the gut that is separate from type 1 pili.^12^ However, studies examining the localization of whole bacteria expressing type 1 or F17-like pili within the mouse gut are required to determine if UPEC bind within the crypts during intestinal colonization in vivo.

The established importance of type 1 pili during infection of the bladder and colonization of the gut served as an impetus for the development of anti-adhesive compounds that target the adhesin FimH. These compounds, called mannosides, are small molecule glycomimetics of the natural host receptor for FimH that display orders of magnitude higher binding affinity, specifically blocking the ability of UPEC to adhere to and colonize the host tissue.^37^ Orally bioavailable mannosides are capable of treating and/or preventing UTI and catheter-associated UTI (CAUTI) in relevant mouse models.^38–40^ Further, these compounds can target and reduce the UPEC intestinal reservoir while simultaneously treating an active bladder infection.^12^ Strikingly, a 16S rRNA study found that oral treatment with mannosides has minimal effects on the overall structure of the gut microbiota, suggesting that mannosides can selectively extirpate UPEC from the gut.^12^

**Fig. 1:**
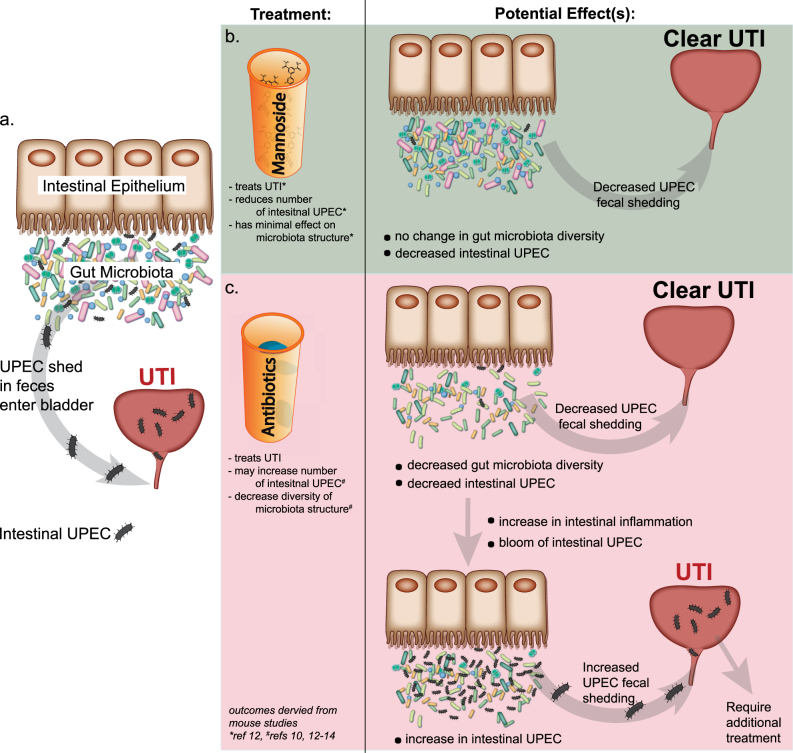
Potential effects of oral mannoside and antibiotic treatment on the intestinal UPEC population. **a** Intestinal UPEC reach the bladder and can cause UTI after being shed in the feces. **b** Oral mannoside treatment targets and reduces the UPEC intestinal population and simultaneously treats and clears UTI in the bladder with minimal effects on the overall structure/diversity of the gut microbiota. **c** Conversely, oral treatment with clinically relevant broad-spectrum antibiotics, like ciprofloxacin, can treat and clear UTI but reduces the overall abundance and diversity of the gut microbiota. The resulting intestinal inflammation caused by antibiotic treatment may promote intestinal *E. coli* colonization (including UPEC) and thus can lead to increase UPEC fecal shedding, promoting recurrent UTI

For some patients, the frequency and severity of rUTIs necessitate long-term prophylactic antibiotic treatment, greatly affecting their quality of life.^41^ Further, withdrawal of the broad-spectrum antibiotic therapy often results in additional UTIs, potentially due to the increase in intestinal UPEC caused by antibiotic-mediated intestinal inflammation. This creates a vicious cycle in which broad-spectrum antibiotic therapy can successfully target and clear UPEC from the bladder but oral antibiotic exposure may actually promote a bloom of intestinal UPEC, which can seed rUTI (Fig. 1a, c). Introducing mannoside treatment alone or in tandem with antibiotic therapy may help to break this cycle and allow for clearance of UPEC from the gut and bladder (Fig. 1a, b).

## Precision treatments for intestinal infections

A precision antimicrobial therapy has also been shown to reduce intestinal colonization by adherent-invasive *E. coli* (AIEC) in a mouse model of CD. Biopsy studies in humans with CD have found that high levels of intestinal inflammation and/or bacterial dysbiosis cause some patients to express abnormally high levels of CEACAM6, a homotypic adhesion molecule, on their ileal epithelium.^42–45^ CEACAM6 is highly mannosylated and serves as a receptor AIEC expressing type 1 pili, resulting in an overgrowth of AIEC. While the role of AIEC in the initiation of CD is controversial, it is accepted that the increased abundance of this bacterium serves to promote the constitutive, overexuberant inflammatory response associated with the disease. A recent study found that oral mannoside therapy is capable of targeting and reducing ileal colonization by AIEC in a transgenic mouse model.^46^ This finding suggests that mannoside treatment may help to reduce the severity of disease in CD patients that are colonized by AIEC. This therapeutic rationale is currently being tested in a Phase I clinical trial with the oral, non-bioavailable FimH antagonist EB8018 to treat CD (ClinicalTrials.gov, NCT02998190).

*Clostridium difficile* (*C. diff.*) is another example of an opportunistic infection that is typically triggered by the use of broad-spectrum antibiotics and destruction of the normal gut microbiota. *C. diff*. infections are the leading cause of hospital-acquired diarrhea and can be highly recurrent, resulting in increased exposure to antibiotics. This has led to a large number of antibiotic-resistant isolates worldwide. In fact, more than 500,000 cases are diagnosed each year in the US alone. Antivirulence strategies targeting these infections include monoclonal antibodies and small molecular inhibitors targeting critical *C. diff*. virulence factors, like toxin A (TcdA) and toxin B (TcdB).^47–50^ The action of these proteases results in the release of its glucosyltransferase domain, which irreversibly glucosylates the RhoA family of GTPases, ultimately leading to apoptosis of infected host cells in the gut. The recently identified small molecule inhibitors have shown good efficacy in both in vitro and in vivo studies.

## Precision treatment for respiratory infections

*Streptococcus pneumoniae* and Nontypeable *Haemophilus influenzae* (NTHi) are commensal residents of the human nasopharynx.^51^ However, like UPEC, both organisms are capable of migrating to body sites outside of the nasopharynx and causing infection. During periods of dysbiosis, as occurs during antibiotic exposure, *S. pneumoniae* and NTHi can infect the normally sterile middle ear, causing OM. OM is one of the most common childhood diseases, affecting 75% of children under the age of 3.^52^ After accessing the middle ear, these bacteria form dense biofilm communities that shield the enclosed bacteria from immune cells and antibiotic treatment.^51^ Because of these challenges, bacterial OM is often chronic and recurrent.

Developing precision antimicrobials that target *S. pneumoniae* and NTHi species in the middle ear and/or the nasopharynx may enhance treatment efficacy. However, targeting these organisms at the site of infection with small molecules may be limited by the ability of the compounds to penetrate biofilms. Yet, treating patients with precision antimicrobials may permit clinicians to reduce the population of these organisms in the host reservoir and thus prevent the re-seeding of the middle ear from this reservoir. Identifying genes that promote the establishment and/or maintenance of *S. pneumoniae* and NTHi in the respiratory tract microbiota could potentially identify targets against which small molecule antagonists could be developed. Interestingly, NTHi and *S. pneumoniae* are also associated with other mucosal infections, including sinusitis and conjunctivitis, suggesting that developing treatments to target these organisms in the host reservoir may help to treat and/or prevent a number of infections.

A significant pathogen in the development of pneumonia is the Gram-negative bacterium *Pseudomonas aeruginosa*. Cystic fibrosis patients are particularly vulnerable to acquiring a lung infection of this type. The global prevalence of multidrug-resistant *P. aeruginosa* is quickly rising, increasing the urgency for the development of alternative treatment strategies. While *P. aeruginosa* infections are more prevalent in immunocompromised individuals, this organism is also a common cause of skin and soft tissue infections in patients with burns or serious wounds. It is also known to regularly colonize medical devices such as catheters, and is a frequent cause of CAUTI. Multiple diverse therapeutic strategies have been proposed and pursued which target *P. aeruginosa*, including novel antibiotics and antimicrobials targeting key bacterial virulence factors.^53^ These bacteria, like UPEC, encode adhesins for host–cell recognition and pathogenesis, the most important of which are LecA (PA-IL) and LecB (PA-IIL). However, unlike FimH, these lectins are not associated with pili, but are secreted and soluble proteins. LecA and LecB function as homotetramers and are known to recognize glycoproteins bearing D-galactose and L-fucose or D-mannose epitopes, respectively. They are also both necessary for adhesion and biofilm formation. Both multi-valent and monomeric LecA and LecB glycoside-based galactoside and mannoside antagonists have been rationally designed using similar strategies to those utilized for the development of FimH mannosides. To date, several groups have identified and demonstrated efficacy for promising glycomimetic compounds targeting LecA/B.^54,55^ The company GlycoMimetics has reported the discovery of a dual LecA/B antagonist, GM-1051 which is in preclinical testing to treat and prevent *P. aeruginosa* infection in 2009,^54^ but no additional information has been published since that time.

## Precision treatment for skin infections

The role of *Propionibacterium acnes* in adolescent acne vulgaris has been established for several decades. Depending on the clinical severity of acne, treatment often includes topical and/or oral administration of broad-spectrum antibiotics, typically tetracycline derivatives. Unfortunately, resistance to these antibiotics is beginning to emerge.^56^ However, recent work has demonstrated that natural products from *Staphylococcus epidermidis*, such as succinic acid, can effectively inhibit the growth of *P. acnes*.^56^ Gaining a better understanding of the molecular mechanism by which products like succinic acid inhibit *P. acnes* growth may provide the basis of naturally based small molecules that more effectively target this organism and prevent infection from occurring.

Perhaps the most widely studied skin infection is caused by *Staphylococcus aureus*, and more specifically, methicillin-resistant *S. aureus* (MRSA). Nasopharynx colonization rates of *S. aureus* are over 20% in the general European population, with rates of over 90% in patients with atopic dermatitis and other skin conditions.^57^ When perturbation of mechanical barriers or immunological barriers occur, these colonizing populations can lead to skin infections, abscess formation, respiratory infections, and, in severe cases, sepsis. Indeed, invasive MRSA infections are associated with a mortality rate of approximately 20%.^58^ Recently, investigators discovered a natural compound produced by the commensal strain *Staphylococcus lugdunensis* that is able to selectively remove *S. aureus* from the nasopharynx.^59^ This effect is mediated by a molecule termed lugdunin, a cyclic bactericidal peptide that may function to selectively inhibit bacterial metabolism but whose precise mechanism of action remains unknown.^59^ A number of other promising small molecule inhibitors have been uncovered that attenuate *S. aureus* pathogenesis by inhibiting processes such as bacterial iron-sulfur cluster assembly, RnpA-mediated RNA degradation, lipoteichoic acid synthesis, and sortase activity.^60–63^ These studies provide additional examples of small molecule inhibitors that selectively target bacterial species in a specific niche. While the development of these treatments remains in its infancy, other conditions such as atopic dermatitis and psoriasis, which are also associated with changes in the skin microbiome, may be amenable to probiotic or small molecule-based treatment approaches.

## Challenges of developing precision therapeutics

While precision antimicrobial therapeutics hold great promise to help combat the antibiotic resistance crisis and spare existing broad-spectrum antibiotics, a great deal of work will be required to better understand the role and efficacy of these types of precision therapeutics, including their ability to target and reduce specific pathogens in humans. Furthermore, in addition to the traditional hurdles facing the development of novel pharmaceuticals such as toxicity, bioavailability, and manufacturing scalability, the development of precision antibacterial agents faces its own unique set of challenges.

Development of traditional antibiotics is predicated on the targeting of core bacterial processes that are shared among all strains in a group of bacterial species while avoiding cross-reactivity with host cellular processes. The core processes targeted by broad-spectrum antibiotics are often mediated by genes that are highly conserved between bacterial species, such as the *gyrA* gene, which encodes a target of the fluoroquinolone ciprofloxacin. In contrast, precision antimicrobials are designed to target processes occurring in only a defined subset of pathogens without affecting either the host or beneficial bacteria within the microbiota. Thus, precision antimicrobials must be tailor-made to target each bacterial pathogen within its specific host niche. Extensive knowledge of the various stages of the pathogen lifecycle is required to design therapeutics that will disrupt a critical pathway necessary for the persistence and/or virulence of the pathogen. UPEC represent a good example of this challenge. Despite decades of research, a clear genetic definition of UPEC remains elusive and a recent study showed that there is not a single set of genes in UPEC that are both necessary and sufficient for bladder colonization.^64^ UPEC are genetically diverse and vary significantly in their carriage of putative urovirulence factors.^64^ Thus, a drug that targets a factor present in only a fraction of UPEC strains causing UTI would limit its usefulness. However, an integrated analysis using multiple measures of pathogenesis, distinct mouse models of infection, in vitro measures of virulence gene function, comparative genomics and transcriptomics revealed that activity of type 1 pili was one of the best predictors of pathogenesis in mouse models.^64^ This analysis shows that targets of precision antimicrobials can be identified even in genetically diverse pathogens through an integrated, multi-disciplinary approach.

Additionally, UPEC can persist within host reservoirs outside of the bladder, such as the gut, to seed multiple recurrent infections.^12^ Fortunately, orally bioavailable mannoside FimH antagonists have been shown to be able to reduce the gut reservoir while simultaneously treating a bladder infection. This exemplifies how taking into consideration the pathogen’s lifestyle in both its site of infection as well as in other reservoirs within the host may provide additional therapeutic value to a precision-based medicine by potentially reducing the risk of recurrences.

Although targets of precision antimicrobial therapeutics can be developed through academic research, the development and deployment of these precision antimicrobial therapies requires the engagement of the pharmaceutical industry and the strengthening of collaborative efforts between these two spheres.

## Conclusions

**Fig. 2:**
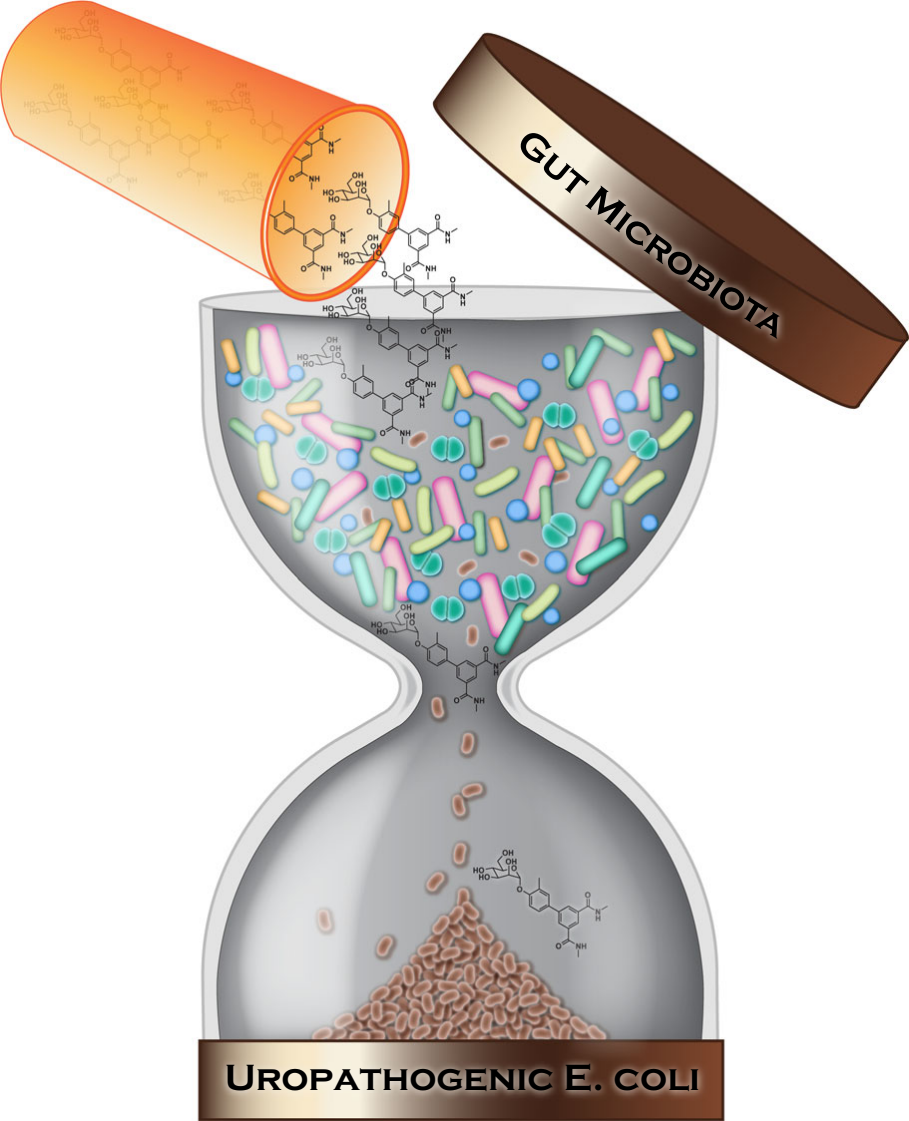
Precision therapeutics target a specific organism while leaving the remainder a microbiota community untouched. Artist rendering of how mannoside treatment (compound from prescription bottle) selectively extirpates UPEC from the gut microbiota

Broad-spectrum antibiotics are invaluable tools for the treatment and prevention of disease; however, the rise of antibiotic-resistant pathogens has made treating individuals with single and multidrug-resistant infections challenging. Further, the increasing number of studies finding that antibiotic-mediated disruption of the microbiota may be detrimental to the host suggests that treating individuals with antibiotics, particularly broad-spectrum antibiotics, has some negative consequences. Therefore, developing precision or “ultra-narrow” spectrum antimicrobials, like mannosides, that are designed to target a specific organism while leaving the remaining microbial community untouched is needed (Fig. 2). Developing therapies that target the host reservoir of pathogens, rather than simply the site of infection, may help to reduce disease burden and/or prevent recurrence.
